# Gut commensal bacterium *Bacteroides vulgatus* exacerbates helminth-induced cardiac fibrosis through succinate accumulation

**DOI:** 10.1371/journal.ppat.1013069

**Published:** 2025-04-16

**Authors:** Jiaqi Wang, Jiali Yin, Xiaolei Liu, Yi Liu, Xuemin Jin

**Affiliations:** 1 State Key Laboratory for Diagnosis and Treatment of Severe Zoonotic Infectious Diseases, Key Laboratory for Zoonosis Research of the Ministry of Education, Institute of Zoonosis, and College of Veterinary Medicine, Jilin University, Changchun, China; 2 College of Animal Sciences, Jilin University, Changchun, China; 3 The Second Hospital of Jilin University, Changchun, China; 4 College of Food Science and Engineering, Jilin University, Changchun, China; Uniformed Services University: Uniformed Services University of the Health Sciences, UNITED STATES OF AMERICA

## Abstract

*Trichinella spiralis* (Ts) is known to cause cardiac fibrosis, which is a critical precursor to various heart diseases, and its progression is influenced by metabolic changes. However, the metabolic mechanisms remain unclear. Here, we observed that Ts-infected mice exhibited cardiac fibrosis along with elevated succinate levels in the heart using metabolomic analysis. Administration of succinate exacerbated fibrosis during Ts infection, while deficiency in succinate receptor 1 (Sucnr1) alleviated the condition, highlighting the role of the succinate-Sucnr1 axis in fibrosis development. Furthermore, metagenomics sequencing showed that Ts-infected mice had a higher abundance ratio of succinate-producing bacteria to succinate-consuming bacteria in the intestines. Notably, the succinate-producer *Bacteroides vulgatus* was enriched in Ts group. Oral supplementation with *B. vulgatus* aggravated Ts-induced cardiac fibrosis. In summary, our findings underscore the succinate-Sucnr1 axis as a critical pathway in helminth-induced cardiac fibrosis and highlight the potential of targeting this axis for therapeutic interventions. This study presents novel insights into the gut-heart axis, revealing innovative strategies for managing cardiovascular complications associated with helminth infections.

## Author summary

Cardiac fibrosis, a pathological stiffening of heart tissue, is a critical precursor to various cardiovascular diseases and can be induced by infections such as those caused by the helminth parasite *Trichinella spiralis* (Ts). This study explores the metabolic pathways that contribute to Ts-induced cardiac fibrosis, with a particular focus on succinate, a key metabolite. Our findings reveal that succinate accumulation in the hearts of Ts-infected mice exacerbates fibrosis. The administration of succinate further intensified this effect, whereas inhibition of the succinate receptor Sucnr1 alleviated the fibrosis. Interestingly, we identified a role for the gut microbiome in this process: Ts-infected mice had increased levels of succinate-producing bacteria, particularly *Bacteroides vulgatus*, which aggravated cardiac fibrosis when supplemented orally. This research highlights the significance of the gut-heart axis in helminth-induced cardiac fibrosis and presents the succinate-Sucnr1 pathway as a potential target for therapeutic intervention. Our study provides novel insights into how gut microbial dysbiosis contributes to cardiovascular complications, emphasizing the therapeutic potential of modulating gut microbiota to prevent or treat heart fibrosis.

## Introduction

Cardiac fibrosis is a pathological condition characterized by the excessive accumulation of extracellular matrix proteins within the heart, leading to stiffening of the cardiac tissue, impaired heart function, and increased risk of heart failure and other cardiovascular diseases [1–4]. It is a critical antecedent to various types of heart diseases and represents a significant factor in the progression of chronic cardiac conditions. Cardiac fibrosis is also a risk factor for severe infection such as viruses and helminths [5–9]. The parasitic worm *Trichinella spiralis* (Ts) is recognized as a significant pathogen responsible for human infectious myocarditis, leading to cardiac fibrosis [10]. A report documenting nine fatal cases of trichinosis revealed that all individuals succumbed to myocarditis associated with fibrosis in the heart [11]. Infection-induced cardiac fibrosis is emerging as a critical global health issue, although the mechanisms by helminths trigger this condition remain poorly understood.

Metabolic alteration is a common process throughout cardiac degeneration [12]. Multiple metabolic pathways can fuel increased collagen synthesis, which is hallmark of cardiac fibrosis [13]. Dysfunction of myocardial energy metabolism including central carbon metabolism is one of the main signs of several cardiovascular diseases [14]. Previously, we found that during the intestinal phase of Ts infection, it can induce cardiac fibrosis [15]. Infections caused by helminths initiate an intricate series of events in the gut microbiome and metabolites that significantly alter the metabolic condition within the gut [16]. The gut microbiome residing in the digestive system impacts well-being through the control of susceptibility to various diseases [17]. The ‘gut hypothesis in heart failure’ has been supported by multiple studies, suggesting that imbalances in the gut microbiota may play a role in negative outcomes for individuals with heart disease [18–21]. The relationship between Ts-altered gut microbiome and metabolism and Ts-induced cardiac fibrosis is of interest.

**Fig 1:**
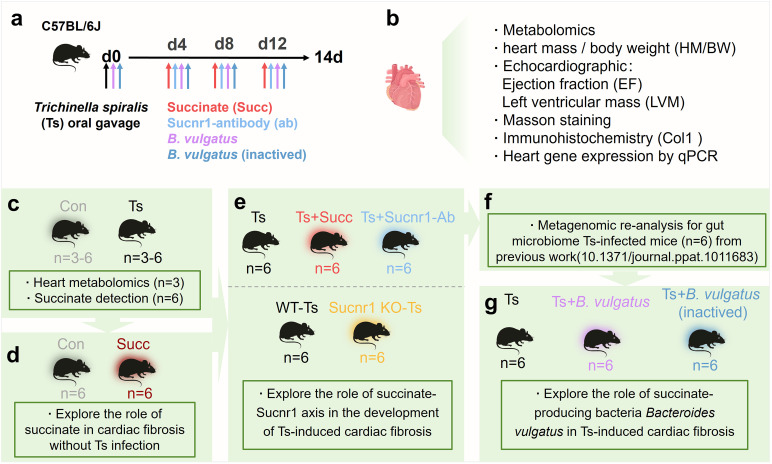
Study design to explore the role of succinate and the succinate-producer *Bacteroides vulgatus* in *Trichinella spiralis* (Ts)-induced cardiac fibrosis in mice. The schematic outlines the experimental timeline and study groups. (a) C57BL/6J mice were orally gavaged with *Trichinella spiralis* (Ts) at day 0 (d0). Various interventions, including succinate (Succ, red), Sucnr1-antibody (ab, light blue), *Bacteroides vulgatus* (purple), and inactivated *B. vulgatus* (dark blue), were administered on days 4, 8, and 12. (b) At day 14, cardiac tissues were collected for metabolomics, heart mass/body weight (HM/BW) analysis, echocardiographic assessment (ejection fraction [EF] and left ventricular mass [LVM]), Masson staining, immunohistochemistry (Col1), and gene expression analysis by qPCR. Experimental groups include: (c) Control (Con, n=3-6) and Ts-infected mice (Ts, n=3-6) for heart metabolomics and succinate detection. (d) Control (n=6) and succinate-treated (n=6) mice to explore the role of succinate in cardiac fibrosis without Ts infection. (e) Ts-infected mice (n=6) with succinate supplementation (Ts+Succ, n=6) and Sucnr1-antibody treatment (Ts+Sucnr1-Ab, n=6). Wild-type (WT-Ts, n=6) and Sucnr1 knockout Ts-infected mice (Sucnr1 KO-Ts, n=6) to assess the role of the succinate-Sucnr1 axis in cardiac fibrosis development. (f) Gut microbiome data from Ts-infected mice (n=6) were re-analyzed from our previous study (DOI: 10.1371/journal.ppat.1011683). (g) Ts-infected mice (n=6) treated with *B. vulgatus* (Ts+ *B. vulgatus*, n=6) or inactivated *B. vulgatus* (Ts+ B. *vulgatus* inactivated, n=6) to investigate the role of succinate-producing bacteria in Ts-induced cardiac fibrosis.

We designed a study incorporating multiple experimental groups (Fig 1). Here, metabolomics showed a higher level of succinate in the hearts of Ts-infected mice. We investigated the role of succinate-Sucnr1 axis in the development of helminth-induced cardiac fibrosis. *Trichinella* causes damage to the intestinal barrier, leading to the entry of some intestinal metabolites into the circulatory system, which has adverse effects on the body. Using metagenomic analyses for gut microbiota, we revealed a unique ratio of abundance of succinate-producing bacteria and succinate-consuming bacteria during Ts infection. Gut succinate-producer *Bacteroides vulgatus* exacerbated the severity of Ts-induced cardiac fibrosis.

## Results

### Succinate level was increased during helminth-induced cardiac fibrosis

**Fig 2:**
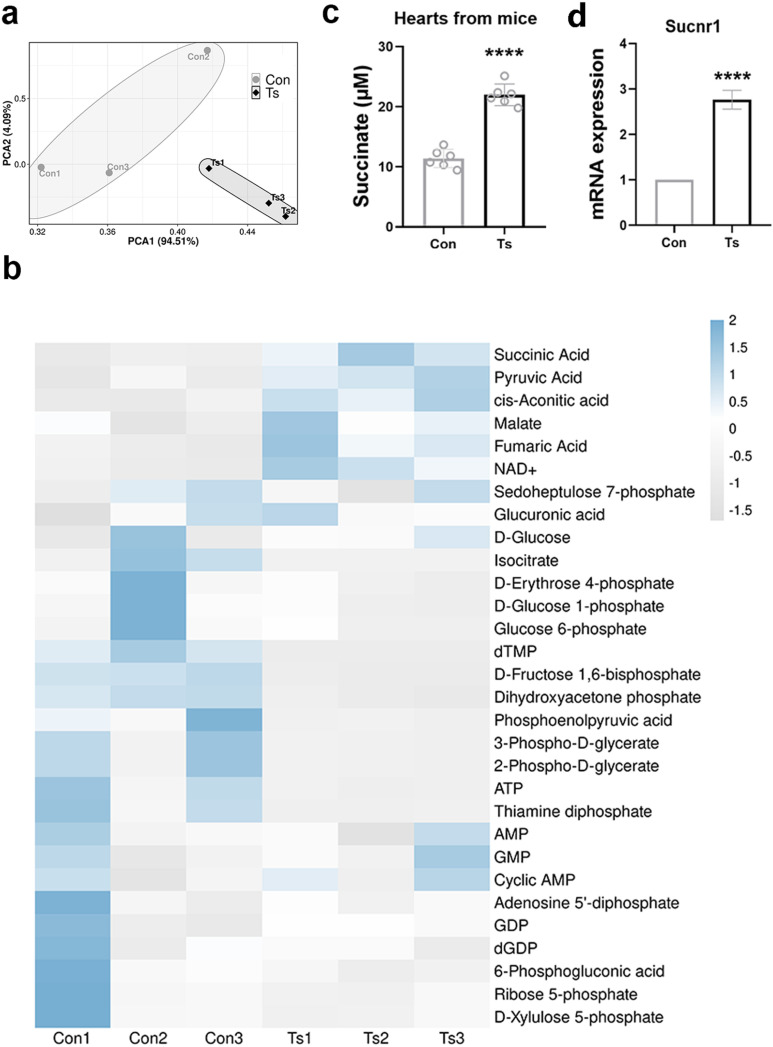
Succinate and Sucnr1 levels were significantly increased. (a) Principal component analysis (PCA) plot (β diversity) (n=3). (b) Cluster heatmap of the differential metabolites in the hearts of mice with or without Ts infection using metabolomic target analyses. n=3 in each group. Statistical significance in metabolomics analysis was determined by analysis of the FDR by the Hochberg-Benjamini test. (c) Succinate levels of hearts from mice (n=6). (d) qPCR analysis of Sucnr1 mRNA expression in the heart tissue of mice (n=6). Data are shown as individual data points and mean ± SD. Statistical significance is calculated using paired student t-test (b and c). ****, p <0.0001.

To measure metabolites from central carbon metabolism during Ts infection, metabolomic target analyses were performed in the hearts from mice. Principal Component Analysis (PCA) was performed to assess the metabolic differences between the control (Con) and infected groups. The PCA plot shows a distinct separation between the Con and Ts samples along the principal components, particularly PC1, which accounts for 94.51% of the total variance. The infected samples (Ts1, Ts2, Ts3) cluster separately from the control samples (Con1, Con2, Con3), indicating marked differences in metabolic profiles following infection (Fig 2A). The heatmap demonstrates relative changes in metabolite concentrations between the control and infected groups. Notably, the levels of succinic acid are significantly (Benjamini–Hochberg false discovery rate (FDR) =0.024) elevated in the infected group compared to the controls (Fig 2B). Other metabolites, such as pyruvic acid, malate, and fumaric acid, also show considerable shifts, indicating broader metabolic reprogramming. A decrease in NAD+ levels was also observed, suggesting potential disruptions in cellular redox balance. The heterogeneity of metabolite profiles within each group is minimal, further supporting the robustness of the observed differences. Quantitative analysis of succinate concentrations in cardiac tissue revealed a significant increase in the Ts group compared to the control group (p < 0.0001), indicating a substantial accumulation of succinate in response to infection (Fig 2C). Succinate receptor 1 (Sucnr1) was identified as a specific receptor for succinate [22]. Gene expression analysis of Sucnr1 in heart tissue showed a marked upregulation in the Ts group compared to the control group (p < 0.0001) (Fig 2D).

### Succinate treatment alone cannot induce cardiac fibrosis

**Fig 3:**
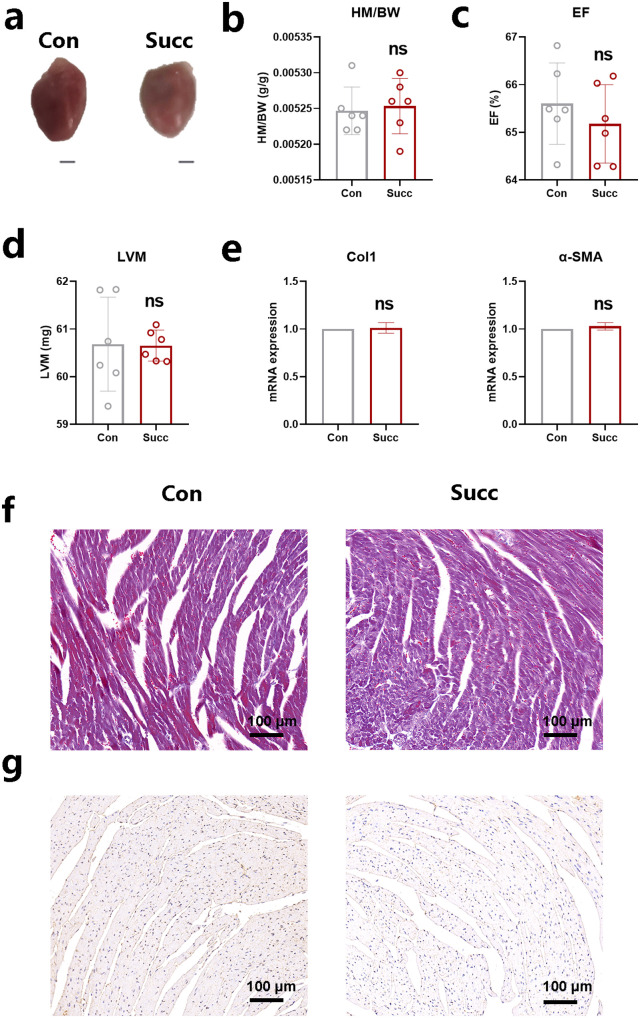
Succinate treatment alone cannot induce cardiac fibrosis. Mice (n=6) under ketamine and xylazine anesthesia had the femoral vein catheterized for intravenous injection of succinate on day 4, 8 and 12. PBS with the same volume were used as control. (a) Whole heart images of mice. Representative images are shown. (b) Heart mass-to-body weight ratio (HM/BW). (c and d) Ejection fraction (EF) and left ventricular weight (LVM) obtained by cardiac ultrasound. (e) qPCR analysis of Collagen-1 (Col1) and α-SMA expression in the heart tissue of mice. (f and g) Masson staining and Col1 immunohistochemistry results of heart tissues. Magnification, 200×. Scale bars, 100 μm. Representative images are shown. Data are shown as individual data points and mean ± SD (n=6). Data were compared by paired student t-test. ns, p > 0.05 compared to the control group (Con).

To investigate whether succinate could induce cardiac fibrosis, mice were treated with succinate alone. The heart mass to body weight (HM/BW) ratio was not changed after succinate treatment (Fig 3A and 3B). There were no differences of ejection fraction (EF) and left ventricular mass (LVM) between the control and succinate-treated mice (Fig 3C and 3D). Collagen-1 (Col1) and α- smooth muscle actin (SMA) are the markers of cardiac fibrosis [23]. Succinate treatment alone did not influence Col1 and α-SMA mRNA expression of hearts (Fig 3E). And when compared to the Ts infection group, succinate administration cannot result in interstitial collagen accumulation, as evidenced by Masson trichrome staining and increased immunohistochemical staining for Col1 (Fig 3F and 3G). These results indicates that succinate treatment alone was not sufficient to cause cardiac fibrosis due to self-adjustment ability in mice.

### Succinate-Sucnr1 axis promoted the development of Ts-induced cardiac fibrosis

**Fig 4:**
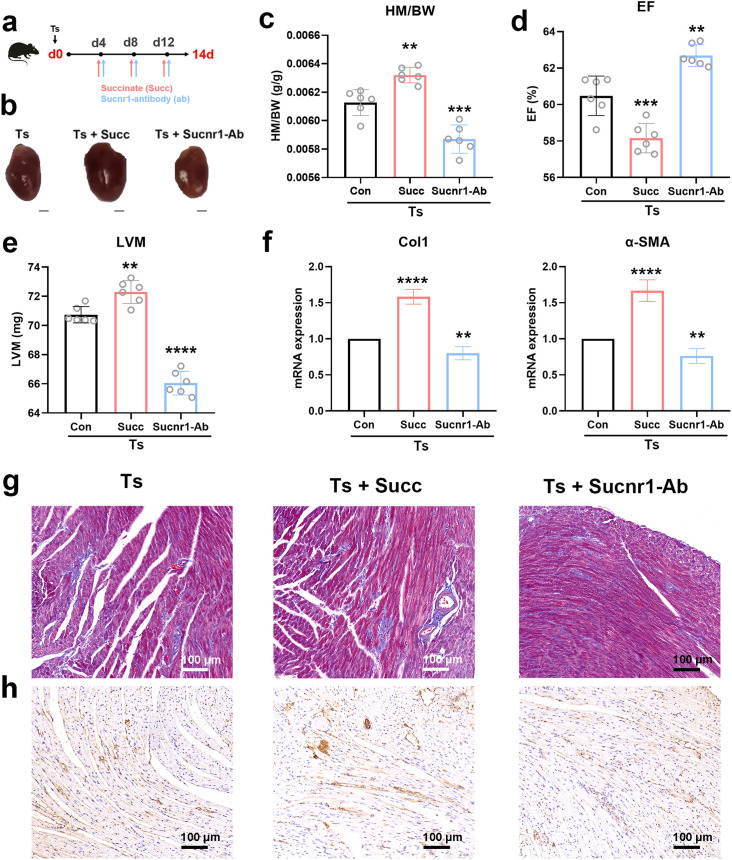
Succinate treatment exacerbated helminth-induced cardiac fibrosis. (a) Experimental scheme of Ts-infected mice received succinate or anti-Sucnr1 antibody. (b) Whole heart images of mice. Representative images are shown. (c) Heart mass-to-body weight ratio (HM/BW). (d and e) Ejection fraction (EF) and left ventricular weight (LVM) obtained by cardiac ultrasound. (f) qPCR analysis of Collagen-1 (Col1) and α-SMA expression in the heart tissue of mice. (g and h) Masson staining and Col1 immunohistochemistry results of heart tissues. Magnification, 200×. Scale bars, 100 μm. Representative images are shown. Data are shown as individual data points and mean ± SD (n=6). Data were compared by one-way ANOVA followed by Tukey multiple comparison tests. **, p < 0.01, ***, p <0.001, ****, p <0.0001 compared to the control group (Con).

**Fig 5:**
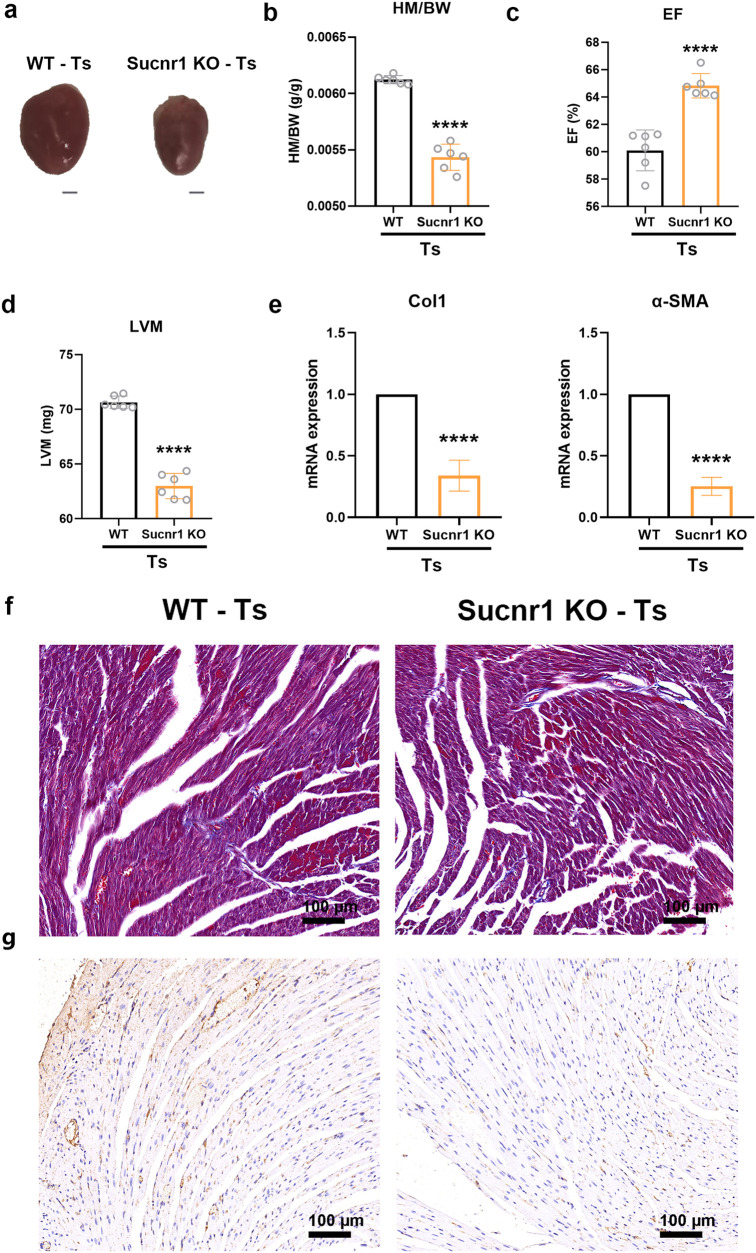
Ts-infected mice deficient in Sucnr1 showed significantly improved cardiac fibrosis. (a) Whole heart images of mice. (b) Heart mass-to-body weight ratio (HM/BW). (c and d) Ejection fraction (EF) and left ventricular weight (LVM) obtained by cardiac ultrasound. (e) qPCR analysis of Collagen-1 (Col1) and α-SMA expression in the heart tissue of mice. (f and g) Masson staining and Col1 immunohistochemistry results of heart tissues. Magnification, 200×. Scale bars, 100 μm. Representative images are shown. Data are shown as individual data points and mean ± SD (n=6). Statistical significance is calculated using paired student t-test. ****, p <0.0001.

To further explore the role of succinate and Sucnr1 in Ts-induced cardiac fibrosis, Ts-infected mice were administered succinate or anti-Sucnr1 antibody (Fig 4A). During Ts infection, succinate administration led to an increased HM/BW ratio (Fig 4B and 4C), a reduction in EF (Fig 4D) and a rise in LVM (Fig 4E). The markers of cardiac fibrosis Col1 and α-SMA mRNA expression were significantly upregulated in the heart following succinate treatment (Fig 4F). When compared to the Ts infection group, succinate administration resulted in greater interstitial collagen accumulation, as evidenced by Masson trichrome staining and increased immunohistochemical staining for Col1 (Fig 4G and 4H). In contrast, neutralization of Sucnr1 could rescue Ts-induced cardiac fibrosis through detecting these cardiac fibrosis-related markers (Fig 4B-4H). Furthermore, there was no difference between Sucnr1 knockout (KO) mice and wild-type (WT) controls (S1A-S1E Fig) without Ts infection. Although Sucnr1 deficiency did not affect the burden of adult worm burden in mice (S2 Fig), Ts-infected mice deficient in Sucnr1 showed significantly improved cardiac fibrosis compared to Ts-infected WT mice (Fig 5A-5G). The results were consistent with those from the above experiment using Sucnr1 neutralizing antibody, suggesting a key role of succinate-Sucnr1 axis in Ts-induced cardiac fibrosis.

### Metagenomic analysis revealed an association of succinate-related bacteria with cardiac fibrosis

**Fig 6:**
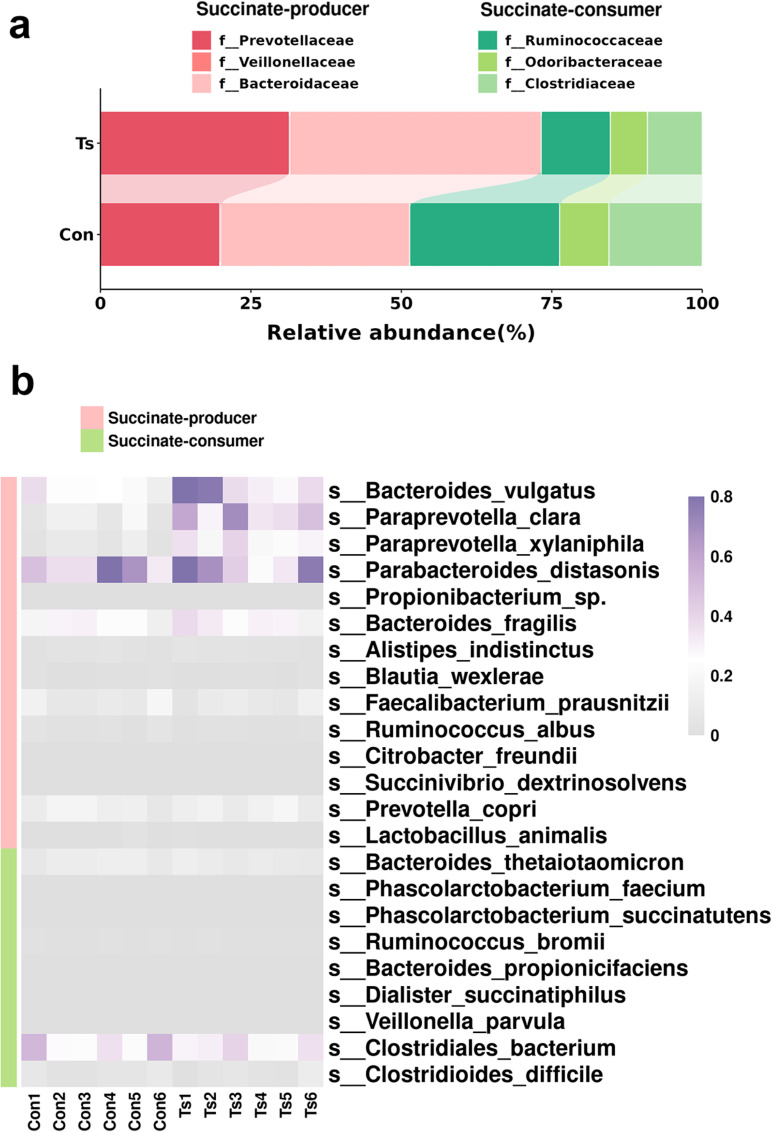
Metagenomic analysis revealed an association of succinate-related bacteria with cardiac fibrosis. (a) Relative abundances of succinate-producer (*Prevotellaceae*, *Veillonellaceae* and *Bacteroidaceae*) and succinate-consumer (*Ruminococcaceae* + *Odoribacteraceae* + *Clostridaceae*). (b) Heatmap of *the* differential succinate-producer and succinate-cosumer in the intestines of mice with or without Ts infection using metagenomics.a Statistical significance was determined by analysis of the FDR by the Hochberg-Benjamini test.

Succinate is notably produced by gut microbiota [24]. To explore succinate-related bacterial species affected by helminth infection, we re-analyzed the gut microbiome in Ts-infected mice using metagenomics, based on our previous research [15]. The results showed that compared to control mice, Ts-infected mice exhibited a higher abundance of succinate-producing bacteria, particularly from the families *Prevotellaceae*, *Veillonellaceae*, and *Bacteroidaceae*, while there was a reduced presence of succinate-consuming bacteria, including those from the families *Ruminococcaceae*, *Odoribacteraceae*, and *Clostridaceae*. (Fig 6A). At the species level, the relative intestinal microbiota including succinate-producer and succinate-consumer [24, 25] were analyzed. Ts-infected mice exhibited a significantly increased abundance of succinate-producers such as *Bacteroides vulgatus* (Hochberg-Benjamini, FDR < 0.0001), *Paraprevotella clara* (Hochberg-Benjamini, FDR < 0.0001) and *Paraprevotella xylaniphila* (Hochberg-Benjamini, FDR =0.0012) (Fig 6B).

### Succinate-producer *B. vulgatus* exacerbated helminth-induced cardiac fibrosis

**Fig 7:**
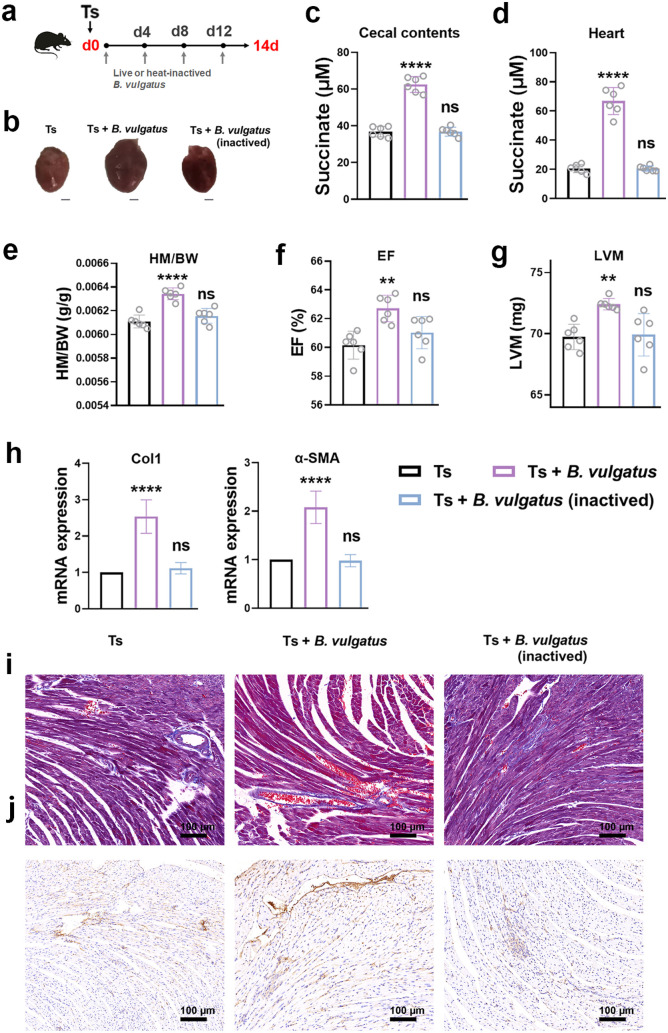
The succinate-producer *Bacteroides vulgatus* exacerbated Ts-induced cardiac fibrosis. (a) Experimental scheme of live or heat-inactived *B. vulgatus* (10^8^ CFU per dose orally) at day 0, 4, 8 and 12 post infection. (b-c) The succinate concentration of cecal contents (b) and hearts (c). (d) Whole heart images of mice. (e) Heart mass-to-body weight ratio (HM/BW). (f and g) Ejection fraction (EF) and left ventricular weight (LVM) obtained by cardiac ultrasound. (h) qPCR analysis of Collagen-1 (Col1) and α-SMA expression in the heart tissue of mice. (i and j) Masson staining and Col1 immunohistochemistry results of heart tissues. Magnification, 200×. Scale bars, 100 μm. Representative images are shown. Data are shown as individual data points and mean ± SD (n=6). Data were compared by one-way ANOVA followed by Tukey multiple comparison tests. **, p < 0.01, ***, p <0.001, ****, p <0.0001 compared to Ts group.

*B. vulgatus* demonstrated in the above results is known to produce succinate [26]. We next determined the role of *B. vulgatus* in the development of cardiac fibrosis. However, *B. vulgatus* supplementation alone cannot induce cardiac fibrosis. Then, oral supplementation with live or heat-inactived *B. vulgatus* were performed after Ts infection as described in Fig 7A. We showed that live *B. vulgatus*, but not inactived *B. vulgatus*, significantly elevated succinate levels in the cecal contents and hearts (Fig 7B and 7C). Live *B. vulgate* could significantly increase the ratio of HM/BW (Fig 7E), decrease EF (Fig 7F) and increase LVM (Fig 7G). The levels of Col1 and α-SMA were significantly increased after live *B. vulgatus* treatment, but not inactived *B. vulgatus* (Fig 7H). Interstitial collagen deposition by Masson trichrome staining and immunohistochemical staining for Col1 were increased after live *B. vulgatus* treatment (Fig 7I and 7J), suggesting that live *B. vulgatus* exacerbated Ts-induced cardiac fibrosis.

## Discussion

Our findings demonstrate that succinate exacerbates the severity of cardiac fibrosis induced by helminth infection. Succinate plays a crucial role in the pathophysiology of various diseases [24]. It has been shown to promote the progression of both lung and liver fibrosis [27–29]. In models of aortic aneurysm and dissection (AAD), a high-risk cardiovascular condition, succinate has been identified as a key factor in disease development and holds potential as a diagnostic biomarker [30]. Succinate acts through its specific receptor, Sucnr1, which has been recognized as vital in regulating immune and metabolic processes [31]. Activation of this receptor by succinate increases its expression in intestinal fibroblasts, and mice lacking Sucnr1 (Sucnr1-/-) are protected from intestinal fibrosis in a model of colonic tissue transplant [32]. The succinate-Sucnr1 signaling pathway is also a key regulator of the molecular mechanisms driving liver fibrosis [29]. Our results further indicate that neutralizing or eliminating Sucnr1 can alleviate helminth-induced cardiac fibrosis, highlighting the critical role of the succinate-Sucnr1 axis in this condition.

Succinate has been shown to trigger a type 2 immune response during infections caused by other helminths, such as *Nippostrongylus brasiliensis*. In this context, succinate promotes tuft cell hyperplasia and expansion of group 2 innate lymphoid cells (ILC2) in the small intestine [33]. Type 2 cytokines, such as IL-13, are known to drive pathological fibrosis across various organ systems [34]. Additionally, Although our current study did not dissect the specific cellular targets of succinate uptake, previous literature indicates that several cardiac cell types express Sucnr1, including cardiomyocytes, fibroblasts, and infiltrating macrophages [35]. Succinate stimulates the production of the pro-inflammatory cytokine IL-1β through the activation of Hypoxia-inducible factor-1α (HIF-1α) in macrophages [36]. IL-1β, in turn, plays a role in promoting cardiac fibrosis [37]. Notably, HIF-1α expression is elevated in failing hearts, and prolonged activation of HIF-1α signaling further contributes to increased fibrosis [38]. Serum IL-1β levels is increased during the enteral stage of Ts infection [39]. Expression of HIF-1α in Ts-infected muscles is increased [40]. However, further mechanistic studies are necessary to clarify the specific roles of HIF-1α and IL-1β in Ts-induced cardiac fibrosis associated with succinate accumulation. Understanding this pathway in greater detail could provide deeper insights into how succinate contributes to fibrosis in this context and help identify potential therapeutic targets for intervention. Moreover, cardiac fibroblasts are pivotal effector cells responsible for extracellular matrix deposition during fibrosis. Succinate binding to Sucnr1 on these cells promotes their activation, proliferation, and differentiation into myofibroblasts, contributing to collagen accumulation and tissue stiffening. Future studies will aim to elucidate specific cell populations through single-cell transcriptomics, cell-specific markers and conditional knockout models.

In addition, helminth infections induce pathological fibrosis across various organ systems [10,34,41], and enteric helminths triggers potent immunomodulation by altering the gut microbiota composition [42–46]. This dysbiosis, along with the resulting disruption of microbiota-related immune processes, plays a critical role in the progression of diseases such as heart disease [47–50]. In fact, depletion of gut microbiota has been shown to worsen outcomes in conditions like myocardial infarction [51]. Our metagenomic analysis observed an increasing abundance of *B. vulgatus* in the intestines of Ts-infected mice. *B. vulgatus* is a commensal gut bacterium and known as succinate-producer [26]. *B. vulgatus* mediates exacerbated inflammation in small intestines [52]. Other study has shown that *B. vulgatus* is closely associated with gut inflammation, with the severity of inflammatory responses being dependent on succinate accumulation [26]. *B. vulgatus* promotes the progression of polycystic ovary syndrome [53], implying that *B. vulgatus* exerts effects at distal locations. Our results suggest that while *B. vulgatus* is not solely responsible for the elevated succinate levels, its heightened activity contributes significantly to succinate accumulation, thereby exacerbating cardiac fibrosis in the context of Ts infection.

Manipulating the microbiome holds significant promise for developing novel treatments for complex human diseases. Succinate, an important intermediate metabolite produced during the breakdown of indigestible dietary fibers and host-derived carbohydrates by gut microbes, is essential in generating short-chain fatty acids [25]. Some bacteria that consume succinate may help regulate immune responses by absorbing excess succinate in intestinal disorders and producing anti-inflammatory compounds. For instance, the transplantation of *Clostridium butyricum* has been shown to mitigate the abnormal rise in intestinal succinate levels caused by antibiotic use [54]. Similarly, probiotic intervention with *Odoribacter laneus* in obese mice helped lower circulating succinate levels [55]. These findings suggest that introducing specific succinate-consuming bacteria could not only decrease excessive succinate but also offer new therapeutic strategies for related diseases. While our data provide compelling correlative evidence linking microbial succinate production with cardiac fibrosis, direct causal evidence remains to be established. Although we did not employ germ-free models, the necessity of microbiota-derived succinate is supported by evidence that depletion of gut microbiota significantly reduces systemic succinate levels and mitigates metabolic disorders [56]. Future investigations that manipulate the gut microbial community—for example, by selectively depleting succinate-producing bacteria or employing succinate-consuming probiotics—would provide further validation of the necessity of microbiota-derived succinate in promoting helminth-induced cardiac fibrosis.

Interestingly, autopsy findings from COVID-19 patients have revealed both diffuse and focal myocardial fibrosis [57,58], suggesting that cardiac fibrosis may significantly contribute to morbidity and mortality in these individuals [59]. Emerging evidence indicates that alterations in the gut microbiota may be linked to the pathogenesis and outcomes of SARS-CoV-2 infection [60]. Metabolomics studies have reported elevated levels of succinate in patients with moderate to severe COVID-19 [61–63], suggesting a potential role for succinate in the disease’s progression. Notably, a case report highlighted that succinylcholine was the primary cause of cardiac arrest in critically ill COVID-19 patients [64]. Succinylmonocholine is hydrolyzed into succinic acid, or succinate. However, data were not gathered to help determine the reason for this relationship. Conducting future investigations to examine the role of succinate on SARS-CoV-2 -associated cardiac fibrosis could provide significant insights.

**Fig 8:**
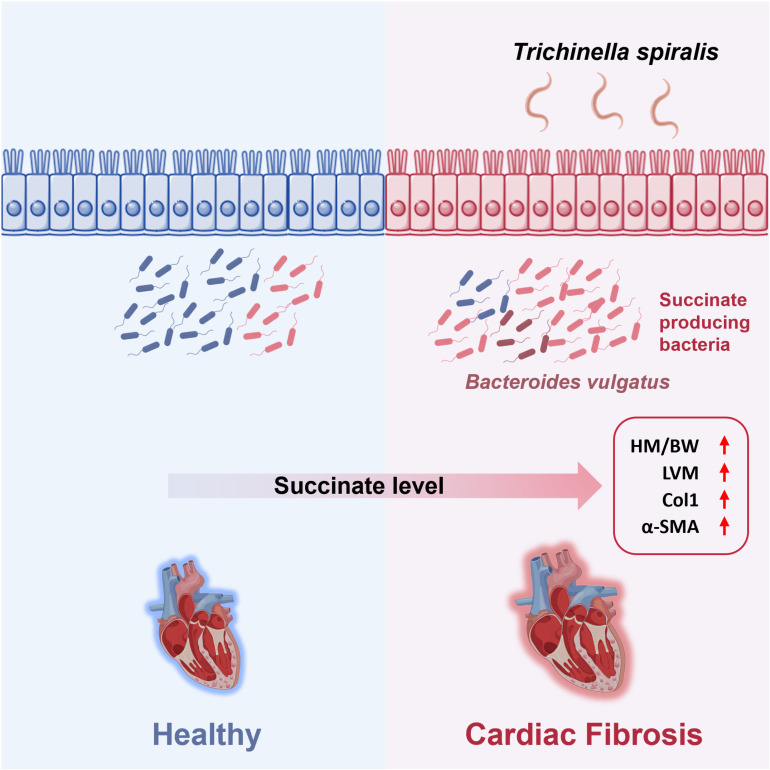
Succinate played a role in helminth-induced cardiac fibrosis. During *Trichinella spiralis* infection, there were more succinate producing bacteria (e.g., *Bacteroides vulgatus*), which increased succinate levels in the intestines and hearts, with an elevated levels of all cardiac fibrosis markers including heart mass-to-body weight ratio (HM/BW), left ventricular mass (LVM), Collagen-1 (Col1) and α-SMA.

In conclusion, this study elucidates the critical role of gut microbiota-derived succinate and its receptor, Sucnr1, in the pathogenesis of helminth-induced cardiac fibrosis. We demonstrate for the first time that the accumulation of succinate, driven by an increased abundance of succinate-producing bacteria such as *B. vulgatus*, significantly exacerbates cardiac fibrosis following helminth infection (Fig 8). This provides new insights into the gut-heart axis and highlights the potential for targeting the succinate-Sucnr1 pathway or modulating the gut microbiota for therapeutic purposes. However, the therapeutic potential of succinate-consuming probiotic bacteria remains to be investigated in future studies.

## Materials and methods

### Ethics statement

All mouse studies and breeding procedures followed the guidelines set forth by the Animal Welfare and Research Ethics Committee of Jilin University. Female C57BL/6J wild-type (WT) mice, aged 4–6 weeks, were procured from Liaoning Changsheng Biotechnology Co., Ltd., China, while female C57BL/6J Sucnr1 knockout (KO) mice of the same age were acquired from Cyagen Biosciences Inc., China. Throughout the experiment, the mice were provided with a standard rodent diet and had unlimited access to water, housed under a 12-hour light/dark cycle. The study protocol received approval from the Institutional Animal Care and Use Committee of Jilin University.

### Helminth infection

*Trichinella spiralis* (Ts) muscle larvae (ML) were harvested from Wistar rats that had been orally infected with 4,000 infective larvae and collected at 35 days post-infection (dpi). To establish a model of helminth-induced cardiac fibrosis, six-week-old male C57BL/6J WT and Sucnr1 KO mice (n=6) were administered 250 TsML via oral gavage. The mice were monitored for weight changes, and data were collected after euthanasia by CO_2_ exposure at 14 dpi.

### Succinate detection

To measure succinate levels in serum, heart tissue, and cecal contents, we utilized a succinate Colorimetric Assay Kit (Sigma–Aldrich, # MAK184). According to the manufacturer’s instructions, in a 96-well plate, add 50 μl sample (or standard) plus 46 µL of Assay Buffer, 2 µL of Enzyme Mix, and 2 µL of Developer, mix gently, and incubate for approximately 30 minutes at 37 °C (protected from light). Measure the absorbance at 450 nm (A_450), and subtract any sample blank readings to obtain corrected values. Use the standard curve to calculate the amount of succinate in each sample.

### Administration of succinate and anti-Sucnr1 antibody

Mice were anesthetized using ketamine and xylazine, after which the femoral vein was catheterized for intravenous injection of either succinate (Sigma Aldrich, #S2378, 0.03 mg/kg) or anti-Sucnr1 antibody (Novus Biological, #NBP1-00861; 2.5 mg/kg) at 4, 8, and 12 dpi. As a control, an equivalent volume of PBS was administered (n=6). All antibodies and solutions were sterilized prior to in vivo administration.

### Oral supplementation of *Bacteroides vulgatus*

*B. vulgatus* ATCC 8482 was purchased from China General Microbiological Culture Collection Center (CGMCC), Beijing, China. *B. vulgatus* was cultured anaerobically in brain–heart infusion (BHI) broth at 37°C using a gas mixture of 5% H_2_, 10% CO_2_, and 85% N_2_. To optimize growth, BHI was supplemented with 0.0005% hemin and 0.5 μg/ml vitamin K1. Mice (n=6) were orally gavaged with live *B. vulgatus* at a dose of 1 × 10^8^ colony-forming units (CFUs) in 0.2 ml of sterile anaerobic PBS at 0, 4, 8, and 12 dpi. As a control, mice received the same dose of heat-killed *B. vulgatus*. Mice were weighed, and after euthanasia with CO_2_ at 14 dpi, serum, cecal contents, and heart tissues were immediately collected and stored at -80°C for further analysis.

### Echocardiographic

Mice underwent transthoracic echocardiography using a high-frequency ultrasound system equipped with an 8-16 MHz probe, following previously established protocols [15].. Mice were anesthetized with 5% isoflurane, which was maintained at 1.25% during imaging. The parasternal short-axis view was obtained at the level of the papillary muscles, and measurements were taken of the left ventricular internal dimensions during diastole and systole (LVIDd/LVIDs) as well as the left ventricular anterior and posterior wall thicknesses (LVAW/LVPW). These parameters were used to calculate the ejection fraction (EF). Both data collection and analysis were performed by an observer blinded to the treatment groups to ensure unbiased results.

### Heart gene expression

The mRNA expression levels of Col1, α-SMA, and Sucnr1 in heart tissue were quantified using real-time polymerase chain reaction (RT-PCR) in accordance with established protocols [15]. Specific primers for these genes are detailed in Table 1. To ensure consistency across samples, expression levels were normalized to the housekeeping gene GAPDH. Relative mRNA levels were then calculated using the comparative Ct method, applying the formula 2^-ΔΔCT^.

**Table 1:** Primers used in this study.

Genes	Primer sequences (5’→3’)
GAPDH-F	TGAAGGGGTCGTTGATGG
GAPDH-R	AAATGGTGAAGGTCGGTGTG
Sucnr1-F	CATATCATGCGCAATTTGAGGA
Sucnr1-R	GCCGTGTCAGTGTGTATATAGA
Col1-F	TCCAAAGGAGAGCGGTAA
Col1-R	GACCAGGACACA
α-SMA -F	TGCTGACAGAGGCACCACTGAA
α-SMA -R	CAGTTGTACGTCCAGAGGCATAG

### Masson staining

Masson’s trichrome staining was performed on 4-µm paraffin-embedded heart tissue sections to assess collagen content, following established methods [15]. The sections were baked at 60°C for 90 minutes, then immersed in xylene three times for 3 minutes each, followed by absolute ethanol twice for 3 minutes each, and 95% and 75% ethanol for 3 minutes each. After rinsing with water, the sections were stained with hematoxylin for 10 minutes, briefly differentiated with hydrochloric acid, and then blued with ammonia for several minutes. They were then stained with Ponceau-acid fuchsin, treated with 12-molybdophosphoric acid, and stained with a green solution for varying times (4-20 seconds, 2-4 minutes, and 2-5 minutes, respectively). After washing with water and baking, the sections were sealed with neutral gum. The stained sections were examined and photographed under a microscope, focusing on five fields of view at 200x magnification for analysis

### Immunohistochemistry

Immunohistochemistry (IHC) was performed on tissue sections using standard protocols. After antigen retrieval and blocking, the slides were incubated overnight with primary antibodies (rabbit anti-mouse Col1a1 at a 1:1000 dilution) in a humidified chamber at 4°C. Rabbit serum served as the negative control. Following PBS rinses, a biotin-labeled goat anti-rabbit secondary antibody was applied for 10 minutes, followed by additional PBS washes. Horseradish peroxidase-conjugated streptavidin was then added and incubated for 10 minutes, with further PBS rinsing. Antibody binding was visualized by staining the sections with 3,3′-diaminobenzidine (DAB) for 1-2 minutes, followed by hematoxylin counterstaining for 1-1.5 minutes. The sections were gently rinsed with water, differentiated in 0.1% hydrochloric acid and alcohol, blued, dehydrated, and cleared using ethanol and xylene. Finally, the sections were sealed with neutral gum and examined under a microscope for imaging

### Metabolomics

The analysis of central carbon metabolism metabolites was conducted by Novogene Co., Ltd. using targeted metabolomics. All of the standards and stable isotope-labeled standards were obtained from ZZ Standards Co., LTD. (Shanghai, China). Methanol (Optima LC-MS), acetonitrile (Optima LC-MS), formic acid (Optima LC-MS) and Ammonium acetate (Optima LC-MS) were purchased from Thermo-Fisher Scientific (FairLawn, NJ, USA). Imino-bis (methylphosphonic acid) was purchased from Sigma-Aldrich (St. Louis, MO, USA). Ultrapure water was purchased from Millipore (MA, USA). In brief, the stock solution of individual CCM related compounds was mixed and prepared in CCM related compounds-free matrix to obtain a series of calibrators. Certain concentrations of D-Glucose-^13^C6、(s)-Malic acid-D3、Succinic acid-D4、dAMP lithium salt-^15^N5 and D-Glucose-6-phosphate disodium salt-^13^C6 were compounded and mixed as Internal Standard (IS). The stock solution of all of these and working solution were stored in refrigerator of -20 °C. The samples (100 mg) were taken respectively and homogenized with 500 μL of methanol/water (4:1) which contained mixed internal standards and set for 5 min. Then centrifuged at 12,000 rpm for 10 min. Finally, the supernatant (2 μL) was injected into the LC-MS/MS system for analysis. An ultra-high performance liquid chromatography coupled to tandem mass spectrometry (UHPLC-MS/MS) system (ExionLC AD UHPLC-QTRAP 6500+, AB SCIEX Corp., Boston, MA, USA) was used to quantitate CCM related compounds in Novogene Co., Ltd. (Beijing, China). Separation was performed on a Waters Atlantis Premier BEH Z-HILIC column (2.1×100 mm, 1.7 μm) which was maintained at 50°C. The mobile phase, consisting of 15mM ammonium acetate with 10 μm imino-bis (methylphosphonic acid) (solvent A) and 15mM ammonium acetate/acetonitrile (solvent B), was delivered at a flow rate of 0.40 mL/min. The solvent gradient was set as follows: initial 95% B, 5 min; 95-70% B, 8min; 70-40% B, 16 min; 40% B, 21 min; 40-95% B, 22.1 min; 95% B, 24 min. The mass spectrometer was operated in negative multiple reaction mode (MRM) mode. Parameters were as follows: IonSpray Voltage (-4500 V), Curtain Gas (35 psi), Ion Source Temp (550 °C), Ion Source Gas of 1 and 2 (60 psi). Data processing involved the use of SCIEX OS Version 1.4 software. Key parameters included a minimum peak height threshold set at 500, a signal-to-noise ratio of 5, and a Gaussian smooth width of 1 to optimize peak detection and reduce background noise. The area under each detected peak was quantified to determine the relative abundance of the corresponding metabolites. Quality control samples and internal standards were included throughout the analysis to ensure reproducibility and data reliability. [65].

Principal Component Analysis (PCA) was performed to assess global metabolic differences among sample groups using the MetaboAnalyst platform (v5.0). The metabolite abundance data were normalized (log-transformation and Pareto scaling) before PCA to reduce bias from highly abundant metabolites. Heatmaps were generated using the pheatmap package in R (v4.1.0) to visualize differential metabolite patterns across samples. Hierarchical clustering was applied using the Euclidean distance metric and complete linkage method. Color gradients were used to represent metabolite abundance, with higher intensity indicating greater abundance.

### Metagenomic sequencing and analysis

For metagenomic sequencing, data were generated from samples associated with the project accession number PRJNA917023 in our previous study [15]. We re-analyzed this data. Raw reads underwent quality filtering where sequencing adapters were removed using cutadapt (v1.9), and low-quality reads were trimmed with fqtrim (v0.94). Quality control of the processed reads was verified using FastQC and Quast to assess metrics such as sequence quality scores, GC content, and duplication levels. To exclude potential host DNA contamination, reads were aligned to the Mus musculus reference genome (GRCm38; accession number GCF_000001635.20) using bowtie2 (v2.2.0), and host-derived sequences were filtered out. The resulting high-quality, non-host reads were assembled de novo using IDBA-UD (v1.1.1), an algorithm optimized for metagenomic assembly of complex microbial communities. Taxonomic classification of clean reads was performed with Kraken2 (v2.1.1), leveraging a k-mer-based approach for rapid classification, followed by abundance estimation with Bracken (v2.5). To achieve detailed taxonomic resolution, unigenes were aligned against the NCBI non-redundant (NR) protein database using DIAMOND (v0.9.14), a fast sequence aligner suitable for large-scale metagenomic datasets.

### Statistical analysis

GraphPad Prism (version 8.0) was used for statistical analyses and graphical representations. To compare two groups, Student’s t-test was employed for continuous variables to determine significant differences. For comparisons across three or more groups, analysis of variance (ANOVA) was performed, followed by Tukey’s post-hoc adjustment for multiple comparisons. Statistical significance in metabolomics and metagenomic analysis was determined by analysis of the FDR by the Hochberg-Benjamini test. Detailed statistical information, including sample sizes (denoted as “n”) for each experiment, is presented in the figure legends. Statistical significance was set at p < 0.05.

## Supporting information

S1 Fig There were no differences between Sucnr1 knockout (KO) mice and wild-type (WT) controls, related to Fig 3. (a) Whole heart images of mice. Representative images are shown. (b) Heart mass-to-body weight ratio (HM/BW). (c and d) Ejection fraction (EF) and left ventricular weight (LVM) obtained by cardiac ultrasound. (e) qPCR analysis of Collagen-1 (Col1) and α-SMA expression in the heart tissue of mice. Data are shown as individual data points and mean ± SD (n=6). Data were compared by paired student t-test. ns, p > 0.05 compared to the control group (Con).
(TIF)

S2 Fig The analysis of adult worm burden in WT and Sucnr1 KO mice, related to Fig 3. Adult worm of *T.spiralis* (Ts) were recovered from mice in each group and the burden of Ts were calculated. Data are shown as individual data points and mean ± SD. Data were compared by paired student t-test. ns, not significant.
(TIF)

S1 Data Excel spreadsheet containing, in separate sheets, the data points presented in Figs 1–6 and S1–S2. (XLSX)
