# Supplementary figures and images for: Gut commensal bacterium Bacteroides vulgatus exacerbates helminth-induced cardiac fibrosis through succinate accumulation
Source: PLoS Pathog. 2025 Apr 16;21(4):e1013069. doi: 10.1371/journal.ppat.1013069 (PMC12002503; doi:10.1371/journal.ppat.1013069)

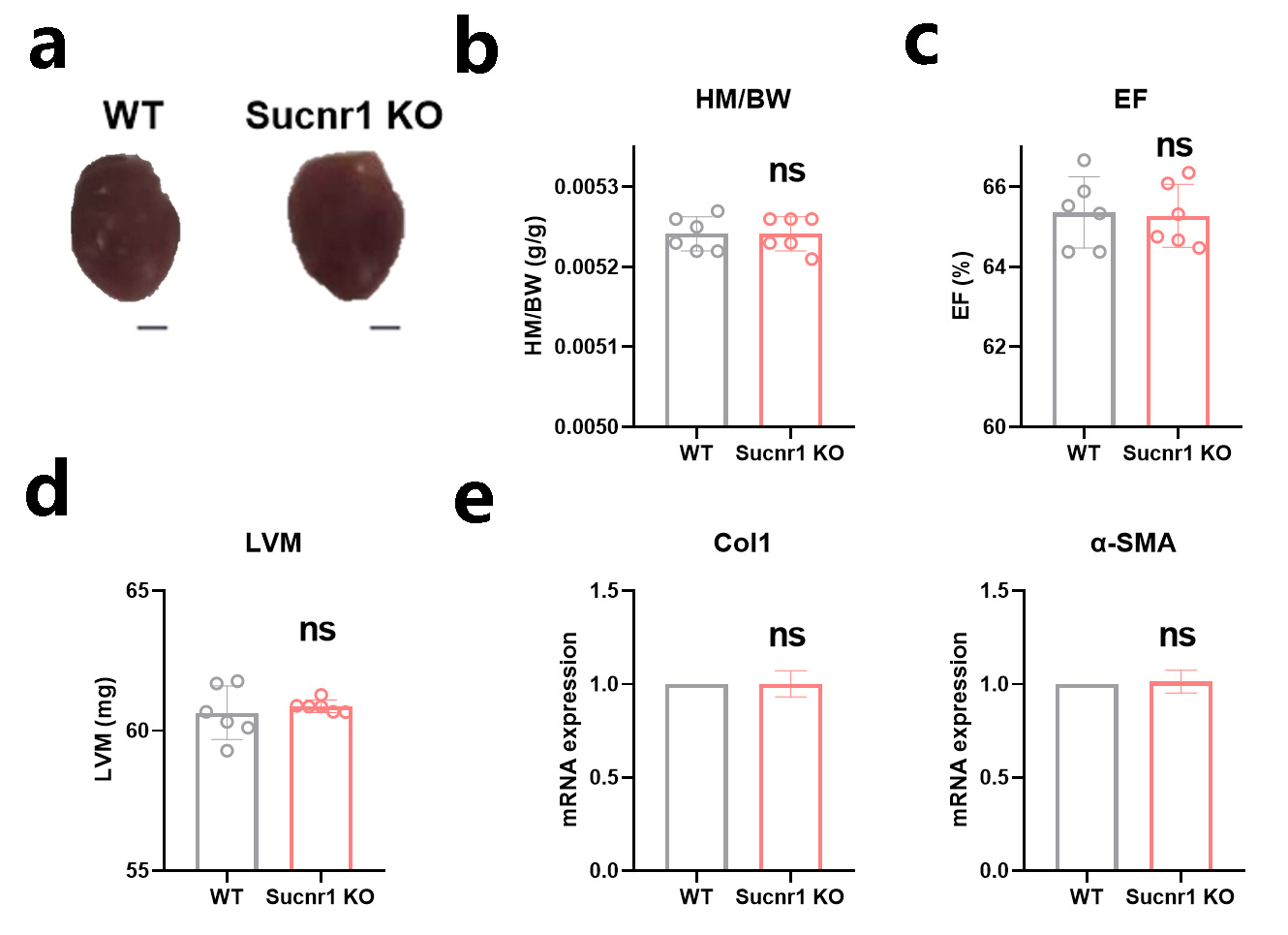

Supplement: S1 Fig — (a) Whole heart images of mice. Representative images are shown. (b) Heart mass-to-body weight ratio (HM/BW). (c and d) Ejection fraction (EF) and left ventricular weight (LVM) obtained by cardiac ultrasound. (e) qPCR analysis of Collagen-1 (Col1) and α-SMA expression in the heart tissue of mice. Data are shown as individual data points and mean ± SD (n=6). Data were compared by paired student t-test. ns, p > 0.05 compared to the control group (Con). (TIF) [file ppat.1013069.s001.tif]

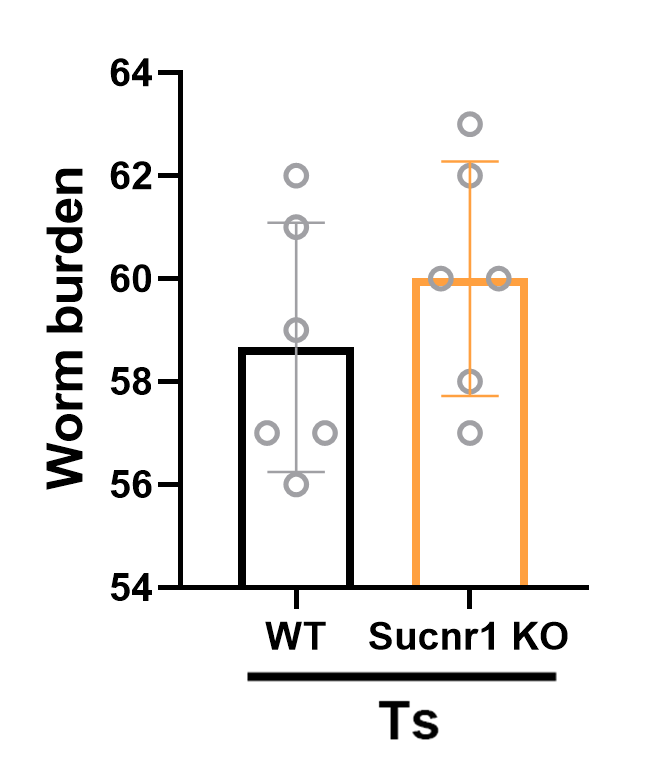

Supplement: S2 Fig — Adult worm of T.spiralis (Ts) were recovered from mice in each group and the burden of Ts were calculated. Data are shown as individual data points and mean ± SD. Data were compared by paired student t-test. ns, not significant. (TIF) [file ppat.1013069.s002.tif]
